# Evaluation and Selection of Stable Reference Genes for qRT-PCR Analysis in Different Tissues of *Mugilogobius chulae* Under Pollutant Exposure

**DOI:** 10.3390/ani16091412

**Published:** 2026-05-05

**Authors:** Zhongdian Dong, Jiahao Gao, Xiaobin Li, Zhishan Chen, Jianjun Li, Jian Liao, Yanping Zhang, Zhongduo Wang, Yusong Guo, Ning Zhang

**Affiliations:** 1 Key Laboratory of Aquaculture in the South China Sea for Aquatic Economic Animal of Guangdong Higher Education Institutes, Fisheries College, Guangdong Ocean University, Zhanjiang 524088, China; 2 Guangdong Provincial Key Laboratory of Aquatic Animal Disease Control and Healthy Culture, Zhanjiang 524088, China; 3 Guangdong Provincial Laboratory Animals Monitoring Center, Guangdong Provincial Biotechnology Research Institute, Guangzhou 510663, China; 4 Key Laboratory of Marine Ecology and Aquaculture Environment of Zhanjiang, Zhanjiang 524088, China

**Keywords:** *Mugilogobius chulae*, reference gene, tissue-specific stability, gene expression normalization, environmental contaminants

## Abstract

Reliable normalization is essential for accurate quantitative real-time PCR (qRT-PCR) analysis, yet suitable reference genes have not been systematically evaluated in *Mugilogobius chulae*, an emerging marine experimental fish used in physiological and environmental research. In this study, 17 candidate reference genes were evaluated in five tissues (brain, gill, gonad, intestine, and liver) of sexually mature male and female *M. chulae* under solvent control (DMSO), bisphenol A (BPA), cadmium (Cd), and sulfamethazine (SMX) treatment conditions. Gene-expression stability was assessed using the comparative ΔCt method, NormFinder, geNorm, BestKeeper, and the integrated RefFinder ranking. The results showed that reference-gene stability was strongly tissue-specific and analysis-dependent. The integrated RefFinder analysis identified *eif3h*, *rpl7*, *rps4x*, *stau1*, and *ef1y* as the most stable genes in the pooled dataset, whereas *ube2*, *hprt1l*, and *aldob* were the least stable. geNorm analysis indicated that two reference genes were sufficient for brain, gill, intestine, and liver, whereas four were required for the gonad and six for cross-tissue comparisons. These findings provide the first systematic basis for reference-gene selection in *M. chulae* and establish an important methodological foundation for future qRT-PCR studies in this species, particularly in research on endocrine disruption, reproductive physiology, and marine ecotoxicology.

## Simple Summary

*Mugilogobius chulae* is an emerging marine model fish with several practical advantages, including small body size, a short life cycle, and ease of laboratory culture. It is increasingly being used in studies of basic biology, physiology, reproduction, and environmental responses. Quantitative real-time PCR (qRT-PCR) is a widely used tool for gene-expression analysis, but reliable results require stable reference genes for normalization. In this study, 17 candidate reference genes were evaluated in five tissues of sexually mature *M. chulae*. The results showed clear tissue-specific differences in gene stability, and no single reference gene was suitable for all tissues. Several genes, including *eif3h*, *rpl7*, and *ef1y*, performed well, whereas *aldob*, *hprt1l*, and *ube2* were generally unsuitable. This study provides a systematic evaluation and practical guidance for reference-gene selection in *M. chulae* and offers practical support for future molecular and physiological research using this promising marine model fish.

## 1. Introduction

*Mugilogobius chulae* is a small warm-water benthic goby widely distributed in estuarine and brackish waters of Southeast Asia and the western Pacific [1]. Although it is not a conventional aquaculture species of direct economic value, its small body size, short life cycle, ease of laboratory culture, and strong adaptability to controlled experimental conditions make it particularly suitable for experimental studies [2,3]. With the establishment of artificial breeding techniques and closed laboratory populations, and genomic and transcriptomic resources, *M. chulae* has emerged as a promising marine model fish and has been increasingly applied in studies of physiology, reproduction, metabolism, environmental responses, and related biological processes [3,4,5,6,7].

Quantitative real-time PCR (qRT-PCR) is one of the most widely used methods for gene expression analysis because of its high sensitivity, specificity, and broad dynamic range [8]. However, accurate and reproducible qRT-PCR analysis depends on appropriate assay design and the use of stably expressed reference genes for normalization [9,10]. Although traditional housekeeping genes such as *β-actin*, *gapdh*, and *18S rRNA* have been widely used for this purpose, numerous studies have demonstrated that their expression stability may vary substantially across species, tissues, developmental stages, sexes, and experimental treatments [11,12,13,14,15]. Therefore, the selection and validation of appropriate reference genes are essential prerequisites for reliable transcriptional analysis [9].

This issue is particularly relevant for *M. chulae* and other emerging small marine or estuarine model fishes, for which molecular methodological tools are less standardized than those for classical freshwater model species. Gene-expression studies in *M. chulae* often involve multiple tissues, both sexes, and exposure to chemically distinct environmental stressors, all of which may influence the basal expression of candidate reference genes. Despite the growing use of *M. chulae* in toxicological and physiological research [5,6,7], suitable reference genes for this species under contaminant exposure conditions have not been systematically evaluated. This represents an important methodological gap, because tissue heterogeneity and chemically distinct pollutants may alter reference-gene stability in different ways [11,16,17]. Therefore, directly adopting conventional housekeeping genes or reference genes validated in other fish species may introduce normalization bias and lead to unreliable interpretation of target-gene expression in *M. chulae*.

Therefore, this study aims to systematically evaluate 17 candidate reference genes in the brain, gill, gonad, intestine, and liver tissues of sexually mature male and female *M. chulae* under bisphenol A (BPA), cadmium (Cd), and sulfamethazine (SMX) exposure conditions, and to identify suitable reference genes for normalization both across all samples and within individual tissues. By incorporating different pollutant classes and both sexes, the study was designed to assess reference-gene stability under a broader range of biological conditions and to improve the robustness of the recommended normalization strategies. In addition, the optimal number of reference genes required for different analytical scenarios was determined using geNorm (https://seqyuan.shinyapps.io/seqyuan_prosper/ (accessed on 30 April 2025)) [18,19]. This study provides a basis for accurate gene expression normalization in *M. chulae* and supports future research on endocrine disruption, reproductive physiology, and marine ecotoxicology in this emerging experimental fish.

## 2. Materials and Methods

### 2.1. Ethics Statement

All animal procedures were conducted in accordance with the Guide for the Care and Use of Laboratory Animals and were approved by the Animal Research and Ethics Committee of Guangdong Ocean University (Approval No. 201903003). The study did not involve endangered or protected species.

### 2.2. Fish Breeding and Pollutant Exposure Design

Sexually mature *M. chulae* (18 months old) were obtained from the Guangdong Institute of Biotechnology (Guangzhou, China). The average body length and body weight were 3.44 ± 0.39 cm and 0.97 ± 0.24 g in females, and 3.97 ± 0.35 cm and 1.46 ± 0.30 g in males, respectively. Fish were maintained in a small recirculating artificial seawater system under controlled laboratory conditions, with salinity at 15 ppt, water temperature at 26 ± 1 °C, and a photoperiod of 14 h light:10 h dark.

After a 2-week acclimation period, healthy fish were randomly assigned to four groups: a solvent control group containing 0.1‰ dimethyl sulfoxide (DMSO), a BPA treatment group (200 μg/L), a Cd treatment group (100 μg/L), and an SMX treatment group (1 mg/L). The selected exposure concentrations were chosen with reference to levels previously used in studies of *M. chulae* [6] or related aquatic organisms [20,21,22,23] and were considered suitable for generating transcriptionally responsive but non-lethal experimental conditions for reference-gene stability assessment.

Fish were maintained in 8 L glass aquaria containing 4 L of exposure solution, with eight individuals per tank (four females and four males). Fish were exposed to the corresponding treatments for 7 days, and 80% of the exposure water was renewed every 24 h to maintain relatively stable pollutant concentrations. Water quality parameters were monitored regularly during the exposure period. The pH (7.8 ± 0.4), dissolved oxygen (DO) (6.8 ± 0.5 mg/L), and conductivity (510 ± 5 μS/cm) remained stable throughout the experiment. Fish were fed twice daily with freshly hatched Artemia salina. At the end of the exposure period, the brain, gill, gonad, intestine, and liver tissues were dissected, immediately preserved in RNA stabilization solution, and stored for subsequent genomic DNA and total RNA extraction. Genomic DNA extracted from representative samples was used to assess potential genomic contamination in cDNA and to verify primer amplification specificity during conventional PCR validation. Gonadal samples included both testes and ovaries from sexually mature fish. Overall, each treatment group contained eight fish (four females and four males), and each treatment–sex–tissue combination included four biological replicates (*n* = 4).

### 2.3. Total RNA Extraction and First-Strand cDNA Synthesis

Genomic DNA and total RNA were extracted from each tissue using an RNA/DNA Co-extraction Kit (Cat: R0017M, Beyotime Biotechnology, Shanghai, China) according to the manufacturer’s protocol. Total RNA concentration and purity were assessed using a NanoDrop 2000 spectrophotometer (Thermo Fisher Scientific, Waltham, MA, USA), and RNA integrity was verified by 1.0% agarose gel electrophoresis. First-strand cDNA was synthesized using TransScript^®^ All-in-One First-Strand cDNA Synthesis SuperMix (Cat: AT341-02, TransGen Biotech, Beijing, China) following the manufacturer’s protocol, with equal amounts (1 μg) of total RNA used for each sample. The resulting cDNA was diluted 10-fold with nuclease-free water and stored at −20 °C until use.

### 2.4. Candidate Reference Genes and Primer Validation

Seventeen candidate reference genes were selected from commonly used protein-coding housekeeping genes in fish, representing diverse functional categories, including cytoskeletal structure (*actb2*), protein synthesis and ribosomal function (*rpl7*, *rps4x*, *eif3h*, *ef1y*), protein folding (*hsp90b*), metabolism (*aldob*, *dera*), and other cellular processes. These genes have been widely evaluated as candidate reference genes in previous qRT-PCR studies across different fish species, providing a broad basis for stability assessment under variable experimental conditions [11,12,14,15,24,25,26]. The β-actin homolog included in this study was *actb2*. In contrast, *18S rRNA* was not included because its extremely high abundance and distinct transcriptional characteristics relative to mRNA targets may compromise its suitability for normalization in mRNA-based expression analyses [11,24].

Gene-specific primers (Table 1) were designed in this study using Primer Premier 5.0 (Premier Biosoft International, San Francisco, CA, USA) based on annotated gene sequences obtained from the available *M. chulae* genomic resources [3] and synthesized by GENWIZ (Suzhou, China). Primer specificity was initially evaluated by conventional PCR using a pooled cDNA sample prepared from all experimental samples. Genomic DNA and ddH_2_O were also included as templates to assess potential non-specific amplification and contamination. Each PCR reaction (20 μL) contained 10.0 μL 2×TransTaq^®^-T PCR SuperMix (+dye) (Cat: AS122-01, TransGen Biotech, Beijing, China), 7.0 μL ddH_2_O, 0.5 μL of each primer (10 μM), and 2.0 μL template.

### 2.5. Quantitative Real-Time PCR and Amplification Efficiency Analysis

Amplification performance was evaluated by standard-curve analysis using a 4- or 5-fold serial dilution of pooled cDNA. qPCR was performed in a 10 μL reaction volume containing 5.0 μL 2×PerfectStart^TM^ Green SuperMix (Cat: AQ601-02-V2, TransGen Biotech, Beijing, China), 2.6 μL ddH_2_O, 0.2 μL of each primer (10 μM), and 2.0 μL cDNA template. No-template controls were included in each run. qPCR was carried out on a LightCycler 96 Real-Time PCR System (Roche, Basel, Switzerland) under the following conditions: 94 °C for 30 s, followed by 40 cycles of 94 °C for 5 s, 60 °C for 15 s, and 72 °C for 10 s. Melting-curve analysis was subsequently performed at 95 °C for 10 s, 65 °C for 1 min, and 95 °C for 1 s to confirm amplification specificity. Each reaction was performed in triplicate.

Amplification efficiency (E) was calculated from the slope of the standard curve using the formula: E = (10^(−1/slope) − 1) × 100% [27], and the coefficient of determination (R^2^) was determined accordingly. Primer pairs were considered acceptable when the amplification efficiency (E) ranged from 90% to 110% and the R^2^ was above 0.99, according to commonly used qPCR criteria. Based on the standard curve results, all cDNA samples were diluted 20-fold and used for subsequent qPCR quantification of the 17 candidate reference genes. For amplification-efficiency analysis, each dilution point of the pooled cDNA standard curve was analyzed in technical triplicate. For expression quantification, each individual cDNA sample was analyzed for each candidate reference gene in technical triplicate. The mean Ct value of the three technical replicates was used for subsequent data processing.

### 2.6. Stability Analysis of Candidate Reference Genes

Gene-expression stability was evaluated using averaged Ct values derived from technical-replicate and biological-replicate processing of the qPCR data. Stability analysis was performed at two analytical levels. First, an all-sample pooled dataset was constructed by combining the averaged Ct values across tissues, treatments, and sexes to evaluate overall reference-gene stability under the full experimental framework. Second, tissue-specific datasets were analyzed separately to identify the most suitable reference genes within each tissue type. The detailed data-processing procedure is described in Section 2.7.

Reference-gene stability was performed using RefFinder (http://blooge.cn/RefFinder/ (accessed on 30 April 2025)) [28], which integrates four commonly used algorithms: the comparative ΔCt method, NormFinder, geNorm, and BestKeeper. RefFinder generates a comprehensive final ranking by assigning an appropriate weight to each gene according to the results of the four algorithms and calculating the geometric mean of these weights.

In addition, geNorm (https://seqyuan.shinyapps.io/seqyuan_prosper/ (accessed on 30 April 2025)) was used to determine the optimal number of reference genes required for reliable normalization under different analytical scenarios [18,29]. The pairwise variation value V_n/n+1_ was used to evaluate whether inclusion of an additional reference gene significantly improved normalization accuracy, with 0.15 used as the conventional threshold.

### 2.7. Data Processing and Statistical Analysis

The experimental data structure consisted of four treatment groups, two sexes, and five tissue types. Each treatment group included eight fish, consisting of four females and four males; therefore, each treatment–sex–tissue combination contained four biological replicates (*n* = 4). For each candidate reference gene, each individual cDNA sample was analyzed in technical triplicate. For each individual tissue sample, the mean Ct value of the three technical replicates was first calculated. Then, Ct values from four biological replicates within the same treatment–sex–tissue combination were averaged. These averaged Ct values were used for Ct-distribution analysis, reference-gene stability assessment, and geNorm pairwise variation analysis. Ct-value distributions were summarized descriptively using mean values and standard deviations. The processed Ct values were used for the stability-ranking analyses and geNorm pairwise variation analysis described in Section 2.6.

## 3. Results

### 3.1. Evaluation of Primer Specificity and Amplification Performance

**Figure 1:**
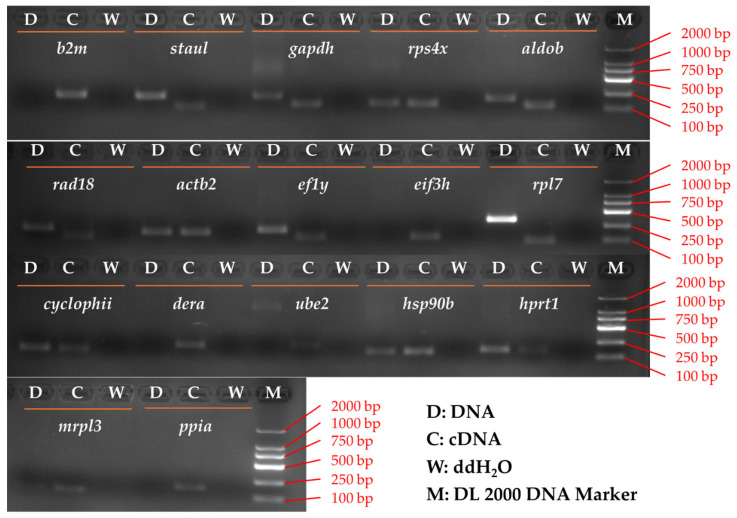
Agarose gel electrophoresis of PCR products of the 17 candidate reference genes. D, genomic DNA; C, pooled cDNA; W, ddH_2_O; M, DL2000 DNA marker. The pooled cDNA template was prepared from all experimental cDNA samples and was used for primer-specificity validation rather than biological comparison.

All 17 primer pairs met the technical requirements for subsequent qRT-PCR analysis, as evidenced by amplicons of the expected sizes, single-peak melting curves, and amplification efficiencies and coefficients of determination within acceptable ranges. Agarose gel electrophoresis confirmed that each primer pair generated a single product of the expected size (Figure 1), and melting-curve analysis further verified amplification specificity (Figure S1). In addition, sequencing and BLAST (https://blast.ncbi.nlm.nih.gov/Blast.cgi (accessed on 30 April 2025)) analysis confirmed that the amplified fragments corresponded to the intended target genes (Table S1). The amplicon lengths ranged from 91 to 194 bp, the amplification efficiencies ranged from 90.2% to 104.3%, and the R^2^ ranged from 0.9901 to 0.9998 (Table 1; Figure S2), indicating that all primer pairs were suitable for further expression-stability analysis.

### 3.2. Expression Profiles of Candidate Reference Genes

**Figure 2:**
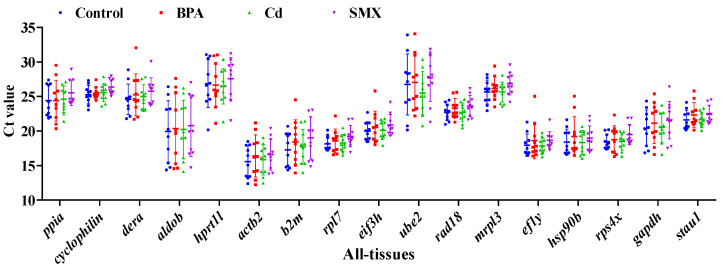
Distribution of Ct values of the 17 candidate reference genes in five tissues under control, BPA, Cd, and SMX treatment conditions. Each point represents the mean Ct value calculated from four biological samples within the same treatment–sex–tissue combination (*n* = 4), after averaging the technical triplicates for each individual tissue sample. In total, 40 averaged Ct values were included for each candidate gene (4 treatments × 2 sexes × 5 tissues). Horizontal lines indicate the overall mean Ct value across all samples for each gene, and vertical bars represent the standard deviation (SD).

The expression levels of the 17 candidate reference genes were assessed in all samples from five tissues under control, BPA, Cd, and SMX treatments. As shown in Figure 2, the Ct values varied substantially among candidate genes, indicating clear differences in transcript abundance and overall variation patterns across samples. The average Ct values ranged from 12.38 for *actb2* to 33.93 for *ube2*. Among the 17 genes, *actb2* and *b2m* showed relatively low Ct values, indicating high transcript abundance, whereas *ube2*, *mrpl3*, and *hprt1l* exhibited relatively high Ct values, indicating lower expression levels.

The dispersion of Ct values was defined as the observed spread or range of the averaged Ct values across all samples for each candidate gene and was used as an initial descriptive indicator of expression variability. Based on this criterion, *rpl7* and *stau1* showed relatively narrow Ct ranges across the averaged sample groups, indicating lower variability, whereas *ube2* and *aldob* displayed broader Ct distributions, suggesting greater variation in expression levels. Moreover, within the same tissue, Ct values under different pollutant treatments showed relatively limited variation compared with the differences observed among tissues (Figure S3), indicating that tissue type contributed substantially to the variation in reference gene expression.

### 3.3. Stability Assessment of Candidate Reference Genes

The expression stability of the 17 candidate reference genes was evaluated in both the pooled dataset and each tissue-specific dataset using four commonly used algorithms: the comparative ΔCt method, NormFinder, geNorm, and BestKeeper. For each individual tissue sample, the mean Ct value of three technical replicates was first calculated. Then, within each treatment–sex–tissue combination, Ct values from four biological samples were averaged and used for downstream stability analysis. The pooled dataset was generated by combining these averaged Ct values across all treatments, sexes, and tissues, whereas tissue-specific datasets were analyzed separately. Because the four algorithms are based on different statistical principles, they may yield partially different stability rankings. Therefore, a comprehensive evaluation was performed using RefFinder, which integrates the results of these algorithms by calculating the geometric mean of their rankings to generate a final stability order. Although the rankings differed somewhat among the individual methods, the overall trends were broadly consistent (Table 2; Table S2).

#### 3.3.1. Comparative ΔCt Analysis

According to the comparative ΔCt method, lower mSD values indicate higher expression stability. In the pooled dataset, *eif3h* was identified as the most stable gene, followed by *stau1*, *ef1y*, *rps4x*, and *rpl7*. Tissue-specific analysis showed that *eif3h* was the most stable gene in brain, gill, and liver, whereas *ef1y* ranked first in the gonad and intestine. In contrast, *aldob*, *hprt1l*, and *ube2* were generally ranked among the least stable genes.

#### 3.3.2. NormFinder Analysis

NormFinder ranked candidate genes based on stability values (SV), with lower SVs indicating higher stability. In the pooled dataset, *eif3h*, *stau1*, and *rps4x* were the three most stable genes. Within individual tissues, the top-ranked genes were *actb2*, *eif3h*, and *rps4x* in the brain; *eif3h*, *stau1*, and *dera* in the gill; *mrpl3*, *dera*, and *ef1y* in the gonad; *mrpl3*, *eif3h*, and *ef1y* in the intestine; and *ef1y*, *mrpl3*, and *rpl7* in the liver.

#### 3.3.3. geNorm Analysis

geNorm ranked candidate genes based on the average expression stability value (M), with lower M values indicating greater stability. In the pooled dataset, *rpl7* and *rps4x* were identified as the most stable gene pair, followed by *eif3h* and *stau1*. In tissue-specific analyses, the most stable gene pairs were *rpl7* and *rps4x* in the brain, *eif3h* and *stau1* in the gill, *mrpl3* and *ef1y* in the gonad, *hsp90b* and *stau1* in the intestine, and *rpl7* and *mrpl3* in the liver. By contrast, *aldob*, *hprt1l*, and *ube2* consistently showed relatively poor stability in the pooled analysis.

#### 3.3.4. BestKeeper Analysis

BestKeeper evaluates gene stability mainly based on the SD and CV of raw Ct values. Compared with the other three algorithms, BestKeeper produced partially different rankings. In the pooled dataset and brain, cyclophilin was ranked as the most stable gene, whereas *hsp90b*, *eif3h*, *actb2* and *ef1y* were identified as the most stable genes in the gill, gonad, intestine, and liver, respectively. Notably, in the pooled dataset, only cyclophilin had an SD value below 1, suggesting that expression variability increased markedly when all tissues were analyzed together.

#### 3.3.5. Comprehensive Ranking by RefFinder

To integrate the results from the four algorithms, RefFinder was used to generate a comprehensive ranking based on the geometric mean of the individual rankings. In the pooled dataset, the five most stable genes were *eif3h*, *rpl7*, *rps4x*, *stau1*, and *ef1y*, whereas *ube2*, *hprt1l*, and *aldob* were the least stable (Table 2). Clear tissue specificity was also observed in the comprehensive rankings. In brain, *actb2*, *rps4x*, *eif3h*, and *rpl7* were the most stable genes. In gill, the top-ranked genes were *eif3h*, *stau1*, *dera*, *b2m*, and *mrpl3*. In the gonad, *ef1y*, *dera*, *eif3h*, *cyclophilin*, and *stau1* showed the highest stability. In intestine, *mrpl3*, *ef1y*, *stau1*, *hsp90b*, and *eif3h* ranked highest, whereas in liver, *rpl7*, *ef1y*, *mrpl3*, *eif3h*, and *rps4x* were the most stable genes. These results indicate that no single reference gene was uniformly optimal across all tissues, and that tissue-specific selection is required for accurate normalization in *M. chulae*.

### 3.4. The Optimal Number of Reference Genes for Genorm Analysis

**Figure 3:**
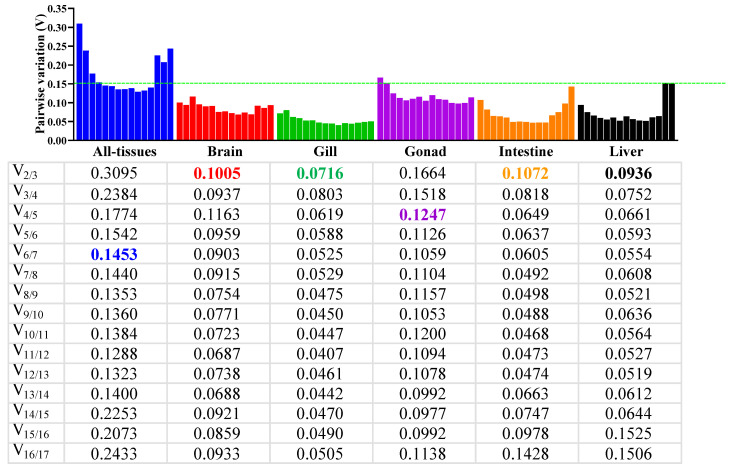
The optimal number of reference genes recommended by geNorm analysis. Pairwise variation values were calculated using the averaged Ct values described in Section 2.7. Each averaged Ct value was derived from four biological samples within the same treatment–sex–tissue combination (*n* = 4) after technical-triplicate averaging. V_2/3_ indicates that two reference genes are sufficient, V_3/4_ indicates that three reference genes are suggested, and so on. V_n/(n+1)_ values below the 0.15 threshold (green dotted line) indicate that n normalization genes are sufficient.

The optimal number of reference genes required for accurate normalization was determined by geNorm pairwise variation analysis using a threshold of 0.15 for V_n/(n+1)_ (Figure 3). In brain, gill, intestine, and liver, the V_2/3_ values were all below 0.15, indicating that two reference genes were sufficient for reliable normalization in each of these tissues. Accordingly, the recommended two-gene combinations were *rpl7* + *rps4x* for brain, *eif3h* + *stau1* for gill, *hsp90b* + *stau1* for intestine, and *rpl7* + *mrpl3* for liver. In the gonad, however, V_2/3_ and V_3/4_ remained above 0.15, and V_4/5_ was the first value to fall below the threshold, indicating that four reference genes were required for more rigorous normalization in this tissue. For the pooled all-tissues dataset, the pairwise variation values remained above 0.15 until V_6/7_, indicating that six reference genes were required when gene-expression comparisons were performed across multiple tissues.

## 4. Discussion

### 4.1. Context-Dependent Stability of Candidate Reference Genes in M. chulae

Accurate normalization is essential for reliable qRT-PCR analysis, particularly in emerging experimental species used in ecotoxicological and physiological studies. *M. chulae* has become an increasingly valuable marine experimental fish because of its small body size, short life cycle, ease of laboratory culture, and suitability for pollutant toxicity assessment [2,3,5,6,7]. However, reference genes suitable for this species under contaminant exposure conditions have not been systematically evaluated. The present study therefore addressed an important methodological gap by assessing the expression stability of 17 candidate reference genes in five tissues of sexually mature *M. chulae* exposed to BPA, Cd, and SMX.

The Ct-value profiles of the candidate reference genes provided an initial overview of their expression patterns under different tissue and treatment conditions. These profiles were not intended to compare pollutant-specific toxicological effects, but rather to evaluate whether candidate reference genes maintained stable expression across different biological contexts. The observed variation in Ct distribution among tissues and treatments indicates that even commonly used housekeeping genes may exhibit expression plasticity under changing experimental conditions [16,17]. This finding further supports the need for systematic validation of reference genes before their use in qRT-PCR normalization.

Reference-gene stability in *M. chulae* was highly context-dependent, which is consistent with previous fish studies demonstrating that no single reference gene is universally stable across tissues or experimental conditions. For example, studies in Atlantic salmon, Atlantic cod, and half-smooth tongue sole have shown that the optimal reference genes can differ among tissues, populations, developmental stages, and chemical-exposure conditions [14,16,17]. In *Pseudocaranx dentex*, *rpl13* and *ef1α* were recommended across adult tissues, whereas *β-actin* and *gapdh* ranked relatively poorly [25]. Similarly, in the multi-tissue study of *Sillago sihama*, several ribosomal protein genes, including rpl7, rpl7a, and rps27, showed comparatively high stability across tissues [30]. Together with the present findings, these studies indicate that reference genes should be empirically validated for each fish species, tissue type, and experimental context rather than selected solely by convention.

### 4.2. Tissue Specificity and Functional Features of Stable Candidates

The strong tissue specificity observed in the present study is biologically reasonable. The brain, gill, gonad, intestine, and liver differ markedly in physiological role and in their expected responses to pollutant stress. The gill is a major interface for waterborne toxicant uptake and ion regulation [31,32], the intestine and liver are central to uptake and xenobiotic metabolism [33,34,35,36], and the brain and gonad are closely linked to neuroendocrine and reproductive regulation [37,38,39]. Under these circumstances, it is unsurprising that genes showing high stability in one tissue may perform less well in another. Similar tissue-dependent patterns have been reported in fish reference-gene studies, such as *P. dentex* [25], where tissue-specific evaluation was required to identify reliable normalization genes. These findings further support the need for tissue-specific rather than universal normalization strategies in fish qRT-PCR studies.

Another noteworthy feature of the present dataset is that several genes associated with ribosomal or translation-related functions repeatedly ranked among the more stable candidates, including *rpl7*, *rps4x*, *ef1y*, and *eif3h*. Similar tendencies have been reported in fish reference-gene studies. In *P. dentex*, *rpl13* and *ef1α* were recommended as stable reference genes across adult tissues [25]. In the multi-tissue study of *Sillago sihama*, *rpl7*, *rpl7a*, and *rps27* also showed comparatively high stability [30]. These observations suggest that genes involved in basic translational and ribosomal processes may provide useful normalization candidates in fish, although their stability still needs to be verified empirically within each species and tissue type.

The selection of candidate reference genes in the present study was guided by commonly used housekeeping genes in fish, while also considering functional diversity. Genes involved in cytoskeletal maintenance, protein synthesis, ribosomal function, protein folding, and metabolism are frequently evaluated as candidate reference genes because they participate in essential cellular processes. However, previous fish studies and the present results indicate that conventional use alone does not guarantee stable expression across tissues, developmental stages, or experimental conditions [14,15,16,17]. These findings support the need to select candidate genes from multiple functional categories and to validate their stability empirically in each fish species and experimental context.

### 4.3. Algorithm-Dependent Rankings and Integrated Evaluation

Partial disagreement among the comparative ΔCt method, NormFinder, geNorm, and BestKeeper was also observed in the present study. This is not unexpected, because the four algorithms are based on different statistical principles: the comparative ΔCt method evaluates pairwise Ct differences [40], NormFinder incorporates intra- and inter-group variation [41], geNorm focuses on average pairwise expression stability [18], and BestKeeper relies mainly on the SD and CV of raw Ct values [42]. Therefore, these methods may produce partially inconsistent rankings. In *P. dentex,* relatively strong agreement among the four methods was observed for *rpl13* and *ef1α* [25], whereas the present study showed greater algorithm-dependent variation for some candidates. These observations support the use of integrated tools such as RefFinder, which help to reduce the bias associated with any single algorithm and provide a more balanced basis for final recommendation.

### 4.4. Optimal Number of Reference Genes Required for Normalization

The present study further indicated that the number of reference genes required for normalization varied markedly among different analytical scenarios. For single-tissue analyses, two genes were sufficient in brain, gill, intestine, and liver, whereas the gonad required four genes; for pooled cross-tissue analyses, six genes were needed before the pairwise variation dropped below the conventional threshold (Figure 3). The higher requirement observed in the gonad may be partly attributable to the intrinsic heterogeneity of the gonadal samples used in this study, which included both testes and ovaries from sexually mature fish. Because these two gonadal types differ substantially in cellular composition, developmental status, and transcriptional background, more robust normalization is likely needed to account for their greater biological variation [43,44,45]. Similarly, the relatively high number of genes required for the pooled dataset likely reflects the greater biological complexity of the experimental design, which combined five tissues, both sexes, and three pollutant classes. This pattern is consistent with the general principle that increased biological heterogeneity requires more robust normalization [18].

### 4.5. Practical Implications, Limitations, and Future Validation

A further point worth noting is that inappropriate normalization can substantially alter the interpretation of target-gene expression, which also highlights the importance of validating selected reference genes whenever possible. In *P. dentex*, the use of *rpl13* and *ef1α*, either individually or in combination, produced consistent *myod1* expression patterns, whereas *β2m*, *β-actin*, and *gapdh* led to marked bias [25]. This example illustrates that statistical stability should ideally be linked to biological reliability through target-gene validation. In this context, a limitation of the present study is the absence of target-gene validation. Although the present work provides a statistically robust basis for reference-gene selection in *M. chulae*, future studies should further verify the recommended combinations using representative target genes involved in physiological regulation, reproductive processes, oxidative stress, xenobiotic metabolism, or other environmentally responsive pathways.

Overall, the present study provides the first systematic evaluation of reference genes for *M. chulae* under pollutant-relevant exposure conditions. Rather than identifying a single universal reference gene, the results provide a tissue-specific normalization framework for qRT-PCR studies in this species. For single-tissue analyses, *rpl7* + *rps4x* can be used as the recommended combination for brain, *eif3h* + *stau1* for gill, *hsp90b* + *stau1* for intestine, and *rpl7* + *mrpl3* for liver, whereas gonadal analyses require a larger reference-gene set because of the combined biological heterogeneity of testes and ovaries. For cross-tissue comparisons, six reference genes are recommended to reduce normalization bias. These recommendations provide a practical starting point for future studies using *M. chulae* to quantify target genes related to environmental response, metabolism, reproduction, or stress physiology. Nevertheless, additional validation is recommended when new developmental stages, tissues, exposure regimes, or target-gene systems are investigated.

## 5. Conclusions

In this study, 17 candidate reference genes were systematically evaluated in the brain, gill, gonad, intestine, and liver of sexually mature *M. chulae* under control, BPA, Cd, and SMX exposure conditions. The results demonstrated that reference-gene stability was strongly tissue-specific and analysis-dependent, and that no single gene was universally suitable across all tissues and treatments. Overall, *eif3h*, *rpl7*, *ef1y*, *rps4x*, *mrpl3*, and *stau1* showed comparatively high stability, whereas *aldob*, *hprt1l*, and *ube2* were generally unsuitable. Two reference genes were sufficient for brain, gill, intestine, and liver, four were required for the gonad, and six were needed for cross-tissue comparisons. These findings provide practical guidance for future qRT-PCR studies in *M. chulae*.

## Figures and Tables

**Table 1:** Genes and primers used in the study.

Gene Symbols	Primer Sequence (5′-3′)	Product Size (bp)	Amplification Efficiency (E) (%)	Coefficients of Determination (R^2^)
*ppia*	F: GCTTCGCTCTGATGTGGTTC R: TGTTTCCGTAGATGGACTTTCC	176	90.2	0.9931
*cyclophilin*	F: GGTTCGCCATTGTTCCACAG R: CAAAGAGCCGCCTACCAAAC	121	101.8	0.9901
*dera*	F: GCCGTCTGCGTTTATCCTTC R: TCTCTGTGGCTCCATCTGCTAC	169	102.3	0.9912
*ube2*	F: ATGATGAGCGGAGACAAAGG R: GTCACAAACTTCACCCGAGG	173	99.6	0.9925
*rad18*	F: TTGTATCCCAACAGATGGCAC R: CGCAGGCATAAGAACTATTTGC	115	99.4	0.9998
*mrpl3*	F: CACCATTCCATAGGTCAGAGTCAC R: GGGTTCCTGGCTTGATGATG	117	99.2	0.9975
*aldob*	F: GCGGCAAACTATTCCCTCAG R: CATCCAAACCTTGTGTTGTGG	120	98.6	0.9929
*hprt1l*	F: TGGACAGGACTGAGCGTTTG R: CAGCGGCACTGATTTATCGC	158	104.3	0.9925
*actb2*	F: GCCTATGTGGCTCTGGATTTC R: ATACCGAGGAAGGATGGCTG	158	97.1	0.9987
*b2m*	F: GTCCTCGTGGCATTGGTCTG R: TCGGTCTGTTCGGAGTCCAG	194	100.1	0.9964
*rpl7*	F: CAACTTCTATGTGCCATCTGAGC R: GCAGGACTTTGCGGACTTTC	92	99.4	0.9971
*eif3h*	F: CATACAGAGGATGATGCGGAC R: AAAGAGCCGTAGTAGGTGGACTG	125	94.3	0.9971
*ef1y*	F: CAACACTTGACCACCCGAAC R: AGTCTGACGGAATGAAGGCTC	120	97.1	0.9996
*hsp90b*	F: CCTTCACCATCCAACCTGTTTC R: GCGAGTGCTTCTTCACAATCTC	129	103.4	0.9934
*rps4x*	F: TGACTGGAGGTGCTAACTTGG R: AGATGTTGGAGAGCCTGGTG	129	98	0.9997
*gapdh*	F: AAATACGACTCCACGCACGG R: CACTTGATGTTGGCTGGGTC	122	94	0.9965
*stau1*	F: AAACGGGCTAACTCATTCACC R: CCGCTACTGCCTACAGCACTAC	91	103.3	0.9986

Note: Forward and reverse primers are indicated as “F:” and “R:”, respectively. Amplification efficiency and R^2^ were calculated from standard curves generated using serially diluted pooled cDNA, with each dilution analyzed in technical triplicate.

**Table 2:** Stability ranking of the 17 candidate reference genes in *M. chulae* based on four algorithms and RefFinder.

Ranking Order (Better-Good-Average)									
	1	2	3	4	5	14	15	16	17
Method	All-tissues								
Delta CT	*eif3h*	*stau1*	*ef1y*	*rps4x*	*rpl7*	*b2m*	*ube2*	*hprt1l*	*aldob*
BestKeeper	*cyclophilin*	*rpl7*	*rad18*	*stau1*	*rps4x*	*b2m*	*hprt1l*	*ube2*	*aldob*
NormFinder	*eif3h*	*stau1*	*rps4x*	*ef1y*	*rpl7*	*b2m*	*ube2*	*hprt1l*	*aldob*
geNorm	*rpl7|rps4x*		*eif3h*	*stau1*	*ef1y*	*b2m*	*ube2*	*hprt1l*	*aldob*
Comprehensive ranking	*eif3h*	*rpl7*	*rps4x*	*stau1*	*ef1y*	*b2m*	*ube2*	*hprt1l*	*aldob*
Method	Brain								
Delta CT	*eif3h*	*actb2*	*rps4x*	*rpl7*	*mrpl3*	*aldob*	*cyclophilin*	*hprt1l*	*b2m*
BestKeeper	*cyclophilin*	*rad18*	*stau1*	*hprt1l*	*aldob*	*hsp90b*	*gapdh*	*ef1y*	*b2m*
NormFinder	*actb2*	*eif3h*	*rps4x*	*rpl7*	*mrpl3*	*aldob*	*cyclophilin*	*hprt1l*	*b2m*
geNorm	*rpl7|rps4x*		*actb2*	*eif3h*	*dera*	*aldob*	*cyclophilin*	*hprt1l*	*b2m*
Comprehensive ranking	*actb2*	*rps4x*	*eif3h*	*rpl7*	*rad18*	*gapdh*	*hsp90b*	*ef1y*	*b2m*
Method	Gill								
Delta CT	*eif3h*	*stau1*	*dera*	*mrpl3*	*b2m*	*rad18*	*ppia*	*ube2*	*hprt1l*
BestKeeper	*hsp90b*	*aldob*	*stau1*	*b2m*	*cyclophilin*	*rps4x*	*hprt1l*	*ube2*	*ppia*
NormFinder	*eif3h*	*stau1*	*dera*	*mrpl3*	*b2m*	*rad18*	*ppia*	*ube2*	*hprt1l*
geNorm	*eif3h|stau1*		*ef1y*	*mrpl3*	*dera*	*rad18*	*ppia*	*ube2*	*hprt1l*
Comprehensive ranking	*eif3h*	*stau1*	*dera*	*b2m*	*mrpl3*	*rad18*	*ppia*	*ube2*	*hprt1l*
Method	Gonad								
Delta CT	*ef1y*	*dera*	*mrpl3*	*eif3h*	*actb2*	*gapdh*	*rpl7*	*hprt1l*	*b2m*
BestKeeper	*eif3h*	*cyclophilin*	*mrpl3*	*rpl7*	*dera*	*actb2*	*hsp90b*	*gapdh*	*hprt1l*
NormFinder	*mrpl3*	*dera*	*ef1y*	*eif3h*	*stau1*	*gapdh*	*rpl7*	*hprt1l*	*b2m*
geNorm	*mrpl3|ef1y*		*dera*	*stau1*	*actb2*	*rps4x*	*rpl7*	*hprt1l*	*b2m*
Comprehensive ranking	*mrpl3*	*ef1y*	*dera*	*eif3h*	*cyclophilin*	*rps4x*	*gapdh*	*b2m*	*hprt1l*
Method	Intestine								
Delta CT	*ef1y*	*eif3h*	*mrpl3*	*stau1*	*aldob*	*b2m*	*ppia*	*ube2*	*hprt1l*
BestKeeper	*actb2*	*rad18*	*mrpl3*	*hsp90b*	*ef1y*	*b2m*	*ppia*	*ube2*	*hprt1l*
NormFinder	*mrpl3*	*eif3h*	*ef1y*	*gapdh*	*cyclophilin*	*b2m*	*ppia*	*ube2*	*hprt1l*
geNorm	*hsp90b|stau1*		*actb2*	*mrpl3*	*ef1y*	*b2m*	*ppia*	*ube2*	*hprt1l*
Comprehensive ranking	*mrpl3*	*ef1y*	*stau1*	*hsp90b*	*eif3h*	*b2m*	*ppia*	*ube2*	*hprt1l*
Method	Liver								
Delta CT	*eif3h*	*rpl7*	*dera*	*mrpl3*	*actb2*	*ppia*	*aldob*	*hprt1l*	*ube2*
BestKeeper	*ef1y*	*rpl7*	*rps4x*	*mrpl3*	*gapdh*	*rad18*	*ube2*	*aldob*	*ppia*
NormFinder	*ef1y*	*mrpl3*	*rpl7*	*eif3h*	*stau1*	*ppia*	*aldob*	*hprt1l*	*ube2*
geNorm	*rpl7|mrpl3*		*ef1y*	*rps4x*	*dera*	*ppia*	*aldob*	*hprt1l*	*ube2*
Comprehensive ranking	*rpl7*	*ef1y*	*mrpl3*	*eif3h*	*rps4x*	*hprt1l*	*ppia*	*aldob*	*ube2*

Note: Ct values used for stability ranking were obtained by first averaging three technical replicates for each individual tissue sample and then averaging four biological samples within each treatment–sex–tissue combination (*n* = 4). The pooled dataset included 40 averaged Ct values for each candidate gene (4 treatments × 2 sexes × 5 tissues), whereas each tissue-specific dataset included 8 averaged Ct values for each candidate gene (4 treatments × 2 sexes). The complete version of this table can be found in Supplementary Table S3.

## Data Availability

The data presented in this study are available on request from the corresponding author.

## Supplementary Materials

The following supporting information can be downloaded at: https://www.mdpi.com/article/10.3390/ani16091412/s1, Figure S1. Melting curves of 17 candidate reference genes. Figure S2. Standard curves of 17 candidate reference genes. Figure S3. Distribution of the Ct values of the 17 candidate reference genes under different treatments. Table S1. Verified sequences for 17 candidate reference genes in the study. Table S2. Stability analysis of 17 candidate reference genes under different conditions calculated by four algorithms. Table S3. Stability ranking of the 17 candidate reference genes in *M. chulae* based on four algorithms and RefFinder (complete version).
