# Supplementary material for: Evaluation and Selection of Stable Reference Genes for qRT-PCR Analysis in Different Tissues of Mugilogobius chulae Under Pollutant Exposure
Source: Animals (Basel). 2026 May 5;16(9):1412. doi: 10.3390/ani16091412 (PMC13163046; doi:10.3390/ani16091412)
Supplement: Supplementary file 1 [file animals-16-01412-s001.zip › Fig.S3.pdf]

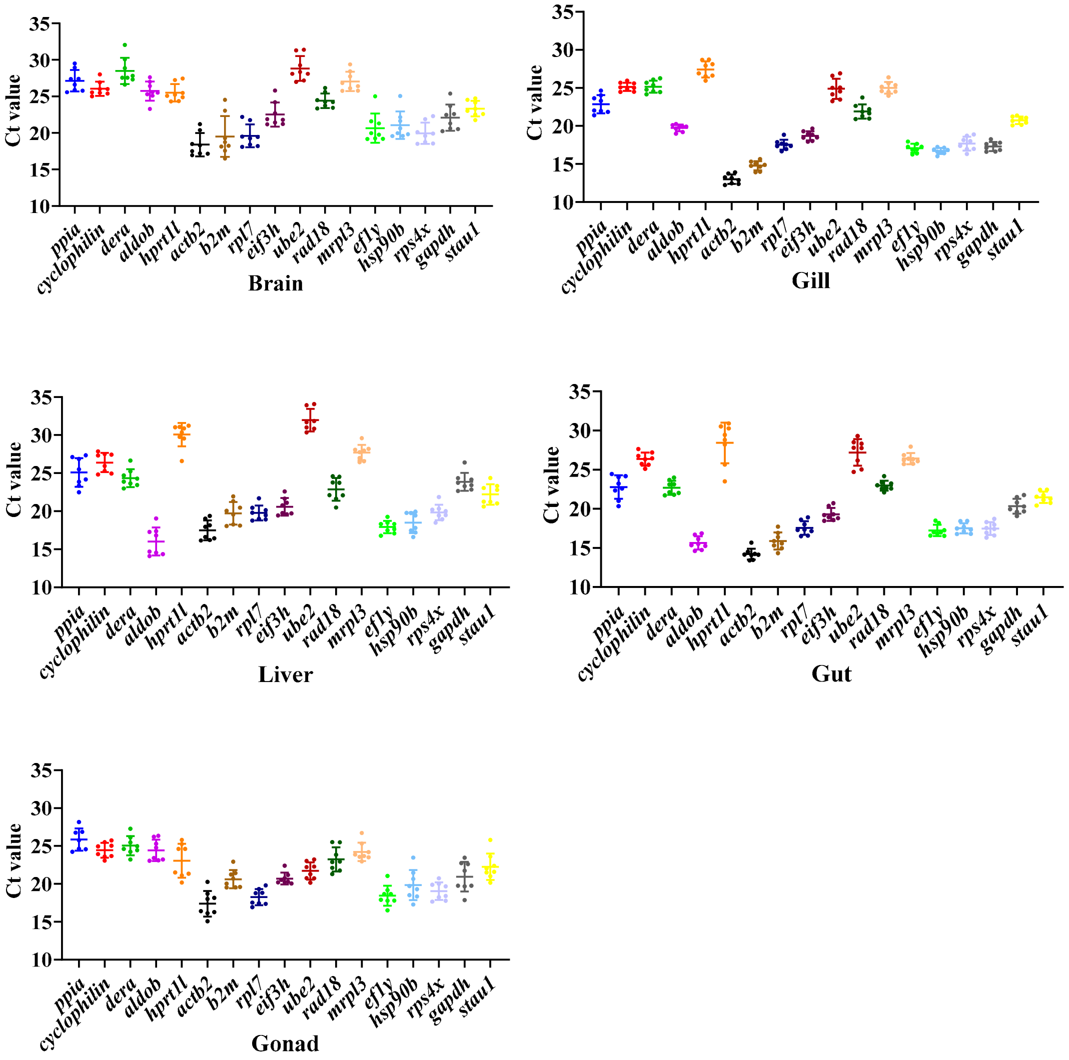

Fig. S3 Distribution of the Ct values of the 17 candidate reference genes under different treatments. Each marker represented the average Ct value of triplicate samples under one tissue at a treatment. The horizontal lines indicate the mean Ct value of all samples under the same conditions. The vertical lines inside standard deviation (SD).
