# Supplementary material for: Evaluation and Selection of Stable Reference Genes for qRT-PCR Analysis in Different Tissues of Mugilogobius chulae Under Pollutant Exposure
Source: Animals (Basel). 2026 May 5;16(9):1412. doi: 10.3390/ani16091412 (PMC13163046; doi:10.3390/ani16091412)
Supplement: Supplementary file 1 [file animals-16-01412-s001.zip › table s1.pdf]

Table S1 Verified sequences for 17 reference genes in the study.

Gene name	Verified sequence
mrpl3	CACCATTCCATAGGTCAGAGTCACAAATGGAGATGTTTAGTAATGCAGG AGTGCCGCCAAAACAGAAAGTCAGCACCTTTAAAGTCTCAGATAATGCC ATCATCAAGCCAGGAACCC
ef1y	CAACACTTGACCACCCGAACCTTCCTTGTGGGAGAGCGAATTAGTCTTGC TGATATCACTGTTGCCTGCTCCATGCTTTGGCTTTACAAACAGGTGCTTG AGCCTTCATTCCGTCAGACT
dera	GCCGTCTGCGTTTATCCTTCCAGAGTTTCGGACGCTGTTAAAACTCTGAG AGCAGCCAACTCCAAACTCCCCGTGGCATCAGTGGCCACAGGTTTTCCA GCAGGACAGACTCATCTCTCCACACGATTGGACGAGGTGCGTTTGGCTG TAGCAGATGGAGCCACAGAGA
eif3h	CATACAGAGGATGATGCGGACTTTGATGAAGTCCAGTACCAGATGGAGA TGATGCGTTCACTGCGTCACGTCAACATTGACCATCTGCATGTGGGCTGG TACCAGTCCACCTACTACGGCTCTTT
cyclophilin	GGTTCGCCATTGTTCCACAGAGAAACGAGGAAGCAAACTTTGATTGTGA CGGACTTTTTATTTCAGAGGAACGGGCGTGGTTGTATTTCATAAGGAAGT GTGTTTGGTAGGCGGCTCTTTG
stau1	AAACGGGCTAACTCATTCACCAAACACATAGGGGTCTTCTCTTTAGGGTT TGCCATGTTTGATTCTGTGGTAGTGCTGTAGGCAGTAGCGG
actb2	GCCTATGTGGCTCTGGATTTCGAGCAGGAAATGAACACTGCTGCCTCCTC CTCCTCTCTGGAGAAGAGCTACGAGTTGCCCGACGGACAGGTCATCACC ATCGGCAATGAGAGGTTCCGTTGCCCAGAGGCTCTCTTCCAGCCATCCTT CCTCGGTAT
rad18	TTGTATCCCAACAGATGGCACAATCATCATCATTAGCTGCAAGCTCTTCA GGCGTGGCAATAGCAAACCTGGCCTCCATGTTGTTAATGACACGCAAAT AGTTCTTATGCCTGCG
hsp90b	CCTTCACCATCCAACCTGTTTCTGGAGAGTCCATTGACCGTGGAACAAAG ATCATTCTGCACTTGAAGGAGGACCAGATGGAGTATGTTGAGGAGAAGA GAATCAAAGAGATTGTGAAGAAGCACTCGC
ppia	GCTTCGCTCTGATGTGGTTCCAAAGACGGCTGAGAACTTCCGCGCTCTCT GCACCGGAGAGAAAGGCTTCGGCTACAAAGGCTGCGTCTTCCACCGCGT CATCACCGAATTCATGTGCCAGGGCGGCGACTTCACAAACCACAATGGA ACCGGAGGAAAGTCCATCTACGGAAACA
ube2	ATGATGAGCGGAGACAAAGGCATCAGTGCTTTTCCTGAATCTGAAAACC TGTTTAAATGGATCGGAACCATCGACGGAGCTCCAGGAACTGTATATGA AGGTCTCCGTTACCGTCTGTCTCTGGAGTTTCCGGCCGGTTATCCGTACC AAGCTCCTCGGGTGAAGTTTGTGAC
aldob	GCGGCAAACTATTCCCTCAGGTCATCAAAGATAAGGGCATTGTAGTCGG TATCAAGGTGGACAAGGGCACAGCTGGGCTCAATGGAACAGACGGGGA AACCACAACACAAGGTTTGGATG

rpl7	CAACTTCTATGTGCCATCTGAGCCCAAACTGGCCTTTGTCATCAGGATCA<br>GAGGTATCAACGGCGTCAGCCCGAAAGTCCGCAAAGTCCTGC
rps4x	TGACTGGAGGTGCTAACTTGGGTCGTATCGGTGTGATCACAAACAGAGA<br>GCGTCACCCCGGATCCTTCGACGTGGTTCACGTGAAGGACAGCACCGGA<br>AACAGCTTCGCCACCAGGCTCTCCAACATCT
gapdh	AAATACGACTCCACGCACGGCCGCTTCAAATGCGGAGAGGTGAAGGCCG<br>AGGGCGGAAAACTCATCATCGGAAACATGTCCATCTCAGTCTTCCAGGA<br>GAGAGACCCAGCCAACATCAAGTG
b2m	GTCCTCGTGGCATTGGTCTGCGCTGTGGAGGCGAAAGAGGCGCGCAATC<br>CTCCCAAGGTGCAACTGTACAGCCGTGATCCCGGTCAGTTTGGCAAGCA<br>AAACACGCTGATCTGCCACGTGAGCGAGTTCCACCCTCCGGACATCACC<br>ATCGAGCTCCTGAAGGATGGAACTCCCCTGGACTCCGAACAGACCGA
hprt1l	TGGACAGGACTGAGCGTTTGGCCCGGGACATCATCCAGGACATGGGAGG<br>ACATCACATAGTAGCCTTGTGTGTGTTAAAGGGAGGCTACAAGTTCTTTG<br>CAGATCTGTTGGACTACATCAAGGCCCTGAACCAGAACAGCGATAAATC<br>AGTGCCGCTG
