# Supplementary figures and images for: Evaluation and Selection of Stable Reference Genes for qRT-PCR Analysis in Different Tissues of Mugilogobius chulae Under Pollutant Exposure
Source: Animals (Basel). 2026 May 5;16(9):1412. doi: 10.3390/ani16091412 (PMC13163046; doi:10.3390/ani16091412)

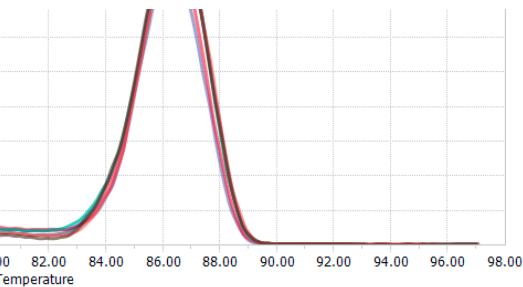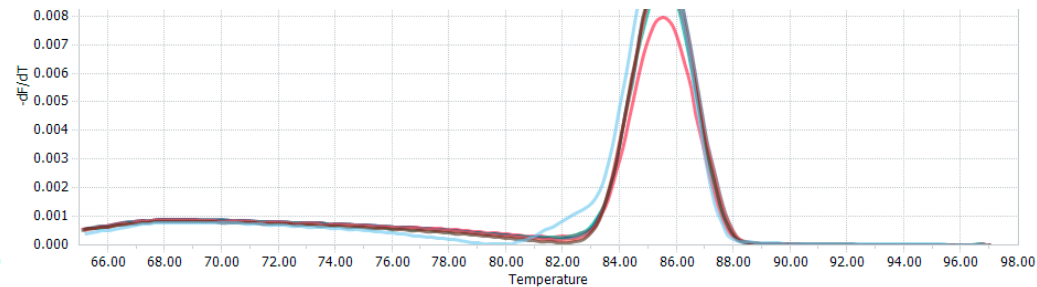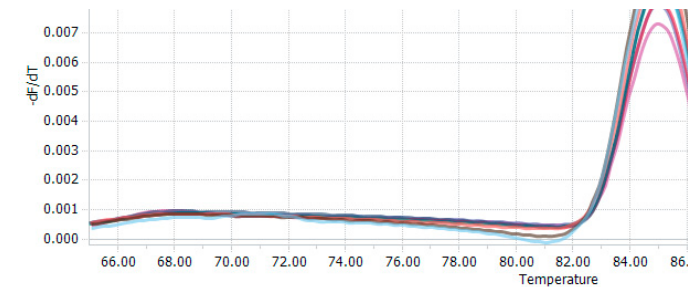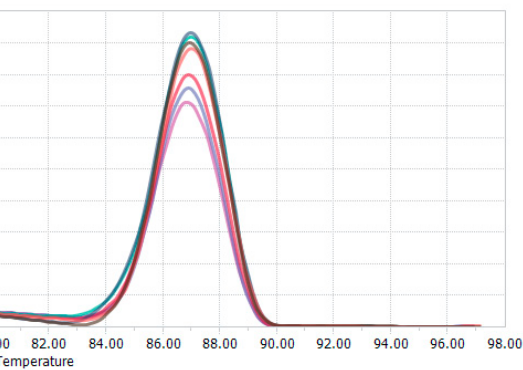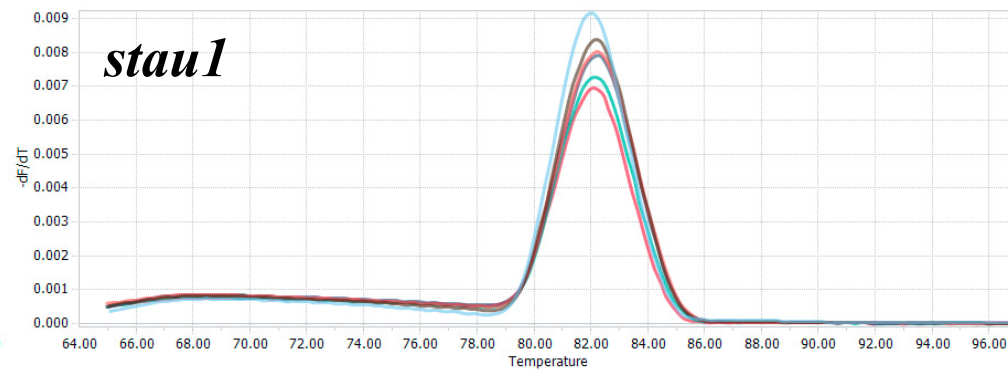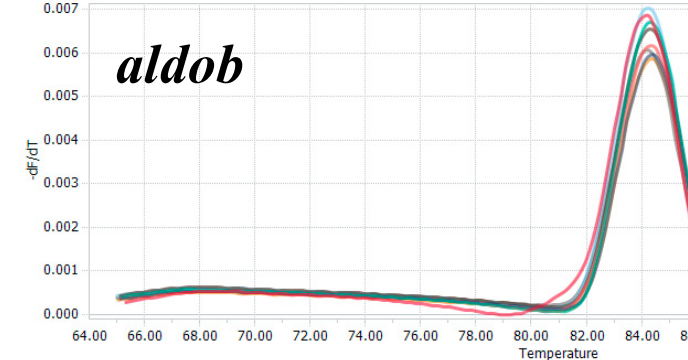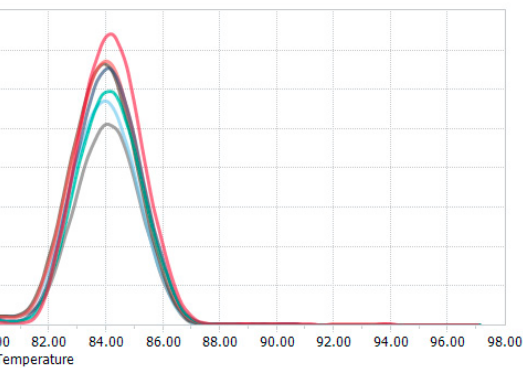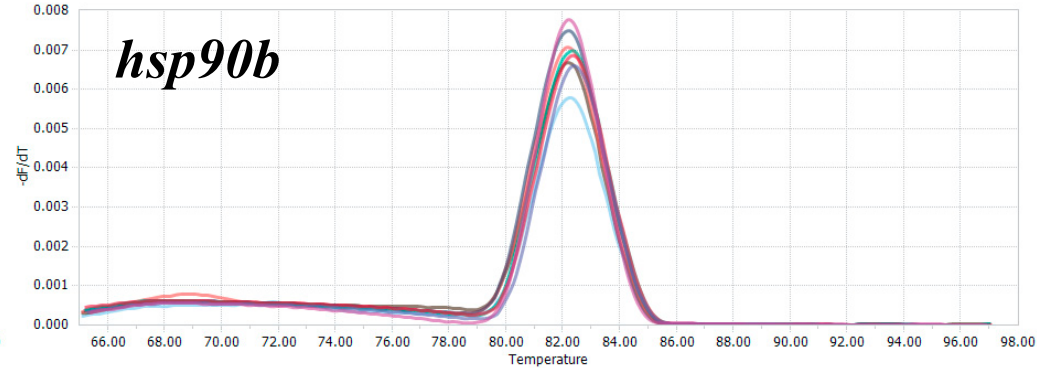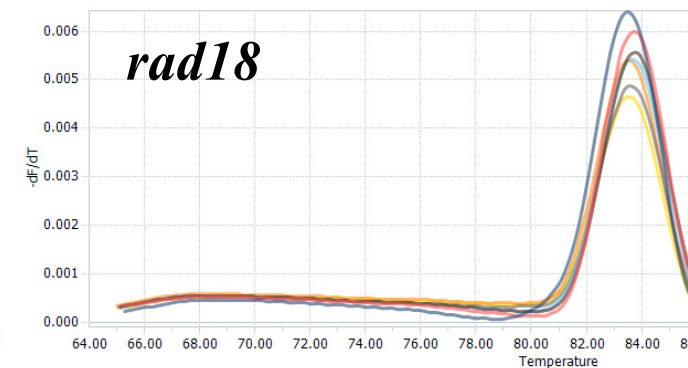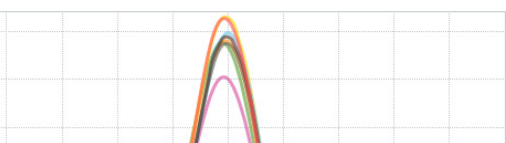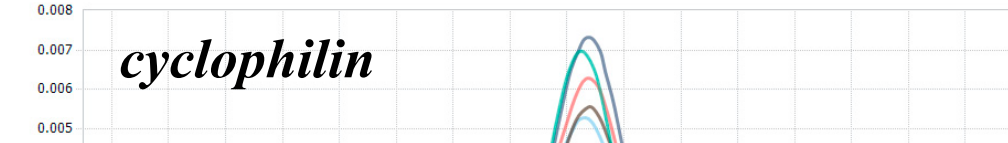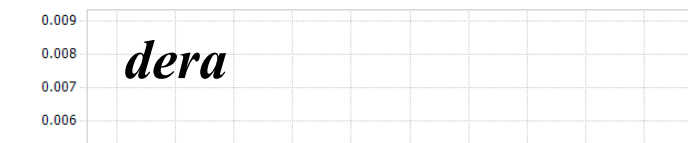

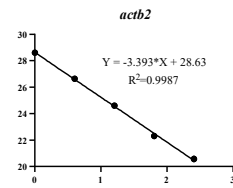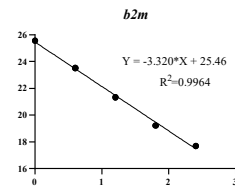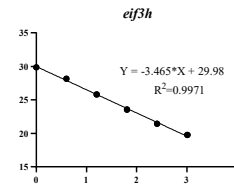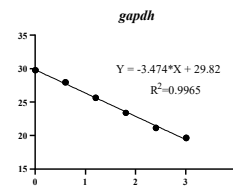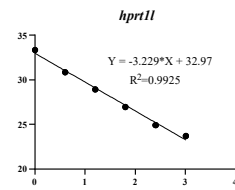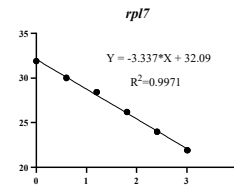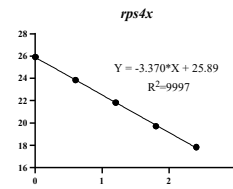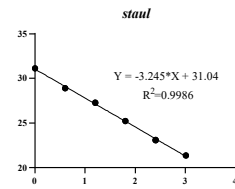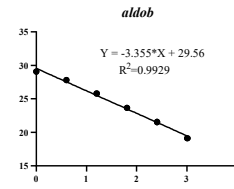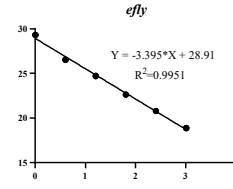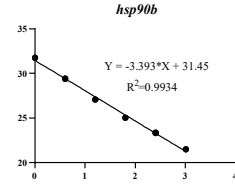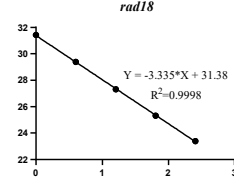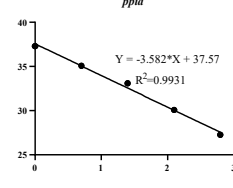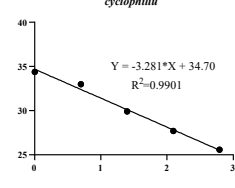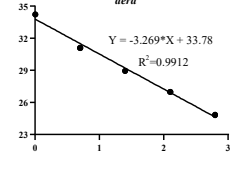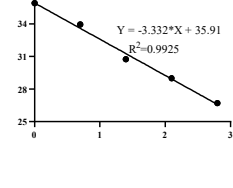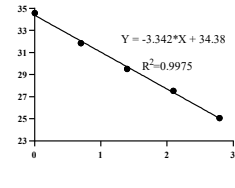

Supplement: Supplementary file 1 [file animals-16-01412-s001.zip › figure S1 and S2.pdf]
